# Revisiting the Genomic Epidemiology of Distinct Phage-Type *Vibrio cholerae* Strains Reveals Restricted Spatiotemporal Dissemination During an Epidemic

**DOI:** 10.3390/microorganisms13071585

**Published:** 2025-07-05

**Authors:** Yu Jiang, Wenxuan Zhao, Xiaorong Yang, Fenxia Fan, Zhenpeng Li, Bo Pang, Biao Kan

**Affiliations:** 1 National Key Laboratory of Intelligent Tracking and Forecasting for Infectious Diseases, National Institute for Communicable Disease Control and Prevention, Chinese Center for Disease Control and Prevention, Beijing 102206, China; jy200879@126.com (Y.J.); fanfenxia@icdc.cn (F.F.); lizhenpeng@icdc.cn (Z.L.); 2 National Institute of Environment Health, Chinese Center for Disease Control and Prevention, Beijing 100021, China; zhao-wx@foxmail.com; 3 Sichuan Center for Disease Control and Prevention, Chengdu 610041, China; yangyangxr@163.com

**Keywords:** cholera, genomics, epidemic, transmission, mutation

## Abstract

The El Tor biotype of *Vibrio cholerae* caused the seventh cholera pandemic (7CP). Although *V. cholerae* variants of this biotype frequently emerge, studies on their microevolution and spatiotemporal transmission in epidemics caused by a single clone are limited. During the cholera outbreak in Sichuan Province, China, in the 1990s, strains belonging to phage type 6 (PT6) but resistant to typing phage VP5 due to a deletion mutation in *ompW*, which is the gene associated with the VP5 receptor were identified. In this study, we analyzed PT6 strains using genome sequencing to reveal the genomic and transmission characteristics of such a transient phage type in China’s cholera epidemic history. The findings revealed that the PT6 strains formed an independent clone during the four-year epidemic and emerged in wave 2. Most of them carried multiple CTX^class^Φ genome copies on chromosome 2 (Chr. 2) and two copies each of RS1^ET^ and RS1-4** on chromosome 1 (Chr. 1). Frequent cross-regional transmission and local outbreaks within Sichuan Province, China, were revealed for this clone. A variety of spontaneous mutations in the *ompW* gene, conferring resistance to the VP5 phage, were observed under VP5 infection pressure, showing the incident mutation of OmpW for the survival adaptation of *V. cholerae* to phage pressure. Therefore, this genomic epidemiological revisit of these distinct phage-resistant phenotype strains reveals their clonal genetic structure, improves our understanding of the spread of *V. cholerae* by tracking their variation, and assists in epidemic source tracing and disease control.

## 1. Introduction

Cholera is an acute and severe dehydrating diarrheal disease. From 1989 to 2023, approximately 200,000–400,000 new cases were reported per year, with a peak exceeding 1.2 million in 2017 [1]. The case fatality rate (CFR) is approximately 2.0% [1]. Significantly, the incidence of cholera fatalities reported in 2023 exhibited a 71% increase compared to 2022, while the number of cases rose by 13% [1]. These numbers may underestimate the actual disease burden due to factors such as limited access to healthcare and insufficient surveillance systems [2]. The etiological agent that causes cholera is *Vibrio cholerae*, which naturally inhabits aquatic environments. The O1 serogroup *V. cholerae* remains the primary cause of cholera and has caused seven cholera pandemics worldwide, with the El Tor biotype *V. cholerae* being the cause of the most recent (seventh) cholera pandemic (7CP). The 7CP broke out in 1961 and was disseminated to various Asian countries before subsequently propagating globally in multiple phases [3,4].

Two major virulence factors affecting the generation of watery diarrhea characteristic of cholera are the cholera toxin (CT) and the toxin-coregulated pilus (TCP). The CT is encoded by the gene *ctxAB*, which is carried by the filamentous bacteriophage CTXΦ. The TCP facilitates the intestinal colonization of the bacterium and acts as a receptor for CTXΦ [5]. An atypical variant of the CTXΦ genome, which lacks the *ctxAB* gene and is referred to as pre-CTXΦ, has been identified and hypothesized to be the precursor of CTXΦ [6,7]. The CTXΦ/pre-CTXΦ genome consists of the core and the RS2 region [5]. The core region includes the *ctxAB* gene together with five other genes (*psh*, *cep*, *orfU*, *ace*, and *zot*). The RS2 element carries the *rstA*, *rstB*, and *rstR* genes. Compared with RS2, RS1 contains an additional gene, *rstC*, which is consistently found in close proximity to CTXΦ in toxigenic El Tor strains. CTXΦ can integrate as one or more copies on chromosome 1 (Chr. 1) or chromosome 2 (Chr. 2) of *V. cholerae* through *attB* sites and form infectious phage particles to infect nontoxigenic *V. cholerae* strains [8]. Hence, the acquisition of CTXΦ played a key role in the evolution of the current pandemic strains [9]. Currently, two genes, *ctxB,* and *rstR*, are commonly employed as proxy indicators for the CTXΦ type [10].

Based on the genomic analysis of the frequency of single-nucleotide polymorphism (SNP) accumulation along with the temporal and geographical distribution of *V. cholerae* strains during transmission, 7CP can be classified into three independent but overlapping waves of global dissemination (wave 1–3) [9]. The strains of wave 1 are defined as prototype El Tor biotype strains carrying CTX-1 (CTX^ET^Φ) with *ctxB3* and the *rstR^ET^* gene on Chr. 1 [9,11]. However, since the mid-1990s, atypical El Tor strains have been isolated predominantly in wave 2 and wave 3. Wave 2 strains generally exhibit a tandem repeat of CTX-2 (CTX^class^Φ) with *ctxB1* and the *rstR^class^* gene on Chr. 2. In addition, certain wave 2 strains also possess CTX^ET^Φ and/or RS1^ET^ on Chr. 1 [11,12]. Early wave 3 strains also carry the *ctxB3* gene, whereas recently isolated strains harbor the *ctxB7* allele [11,13]. These strains still possess the *rstR^ET^* gene but lack components on Chr. 2. Moreover, through the gradual accumulation of mutations, the waves of global dissemination integrate distinct clades that characterize various genetic clones [14,15]. Within these clades, different branches define strains that are phylogenetically and closely related at the genomic level. These strains can be utilized for the epidemiological analyses of transmission during outbreaks, revealing transmission chains and tracing origins.

Over the course of an epidemic, pathogens undergo continuous mutations, with some clones forming that persist in transmission and dissemination. Historically, the characterization of these circulating strains has relied on various biological characteristics, such as serotyping, biotyping, and even phage typing [3]. Compared with genomic sequencing, which allows for the more precise differentiation of these strains and facilitates the investigation of their dissemination patterns, these classification methods, which are based on a limited number of biological traits, provide only a coarse understanding of the information [16]. For example, a phage-biotyping scheme, which is an important tool for the typing of O1 El Tor strains in China, was established in the 1970s [17]. By comparing the lytic profiles of five phages (VP1–VP5) isolated from natural water bodies and fecal samples, El Tor strains were classified into 32 types. During the monitoring period, nearly all El Tor strains were sensitive to all five types of phages according to the phage-biotyping scheme. Consequently, these strains were classified as phage type 1 (PT1) [18]. However, previously unreported strains characterized by resistance to the typing phage VP5 emerged in Sichuan Province in 1998 and were defined as phage type 6 (PT6) [19]. Moreover, PT6 was later found to result from a mutation in the *ompW* gene, which is conserved in *V. cholerae* and encodes the OmpW protein, which is the receptor for typing phage VP5 [18,20]. The same 11 bp deletion corresponding to nucleotides 298 to 308 of the *ompW* gene was detected in PT6 strains, and PT1 strains with an intact *ompW* gene were identified during the same period. Notably, PT6 strains have not been detected outside of Sichuan Province, resulting in distinct spatiotemporal limitations and clear transmission within this region. However, phage typing solely defines strain populations on the basis of phenotypic characteristics, which fail to capture the variations in strains during transmission. Therefore, conducting genomic sequencing enables a detailed analysis of the subtle variations in PT6 strains throughout the transmission process, as well as assisting in accurately delineating the transmission pathways [21,22].

Within a genomic epidemiology framework, we leveraged whole-genome sequencing to dissect genetic variations in PT6 strains, precisely delineating the transmission dynamics of a distinct *V. cholerae* clone across Sichuan Province, China. By tracking and outlining the emergence and transmission of *V. cholerae* with their genome sequences, we identified the clonal expansion of PT6 strains and delineated this from the complex epidemic clones of *V. cholerae*. Thus, we conducted an uninterrupted analysis of the spatiotemporal transmission of a *V. cholerae* clone in Sichuan Province. Moreover, we also observed that *V. cholerae* presented *ompW* gene mutations under typing-phage VP5 pressure, with diverse mutation locations and forms. Identifying and precisely analyzing PT6 strains might yield insights into the genetic variation and transmission dynamics of cholera.

## 2. Materials and Methods

### 2.1. Bacterial Strains, Phages, Plasmids, and Culture Conditions

This research was performed as part of routine infectious disease surveillance and management. A total of 131 O1 toxigenic El Tor *V. cholerae* strains isolated from patients were selected for whole-genome sequencing, including 70 strains belonging to PT6 isolated in Sichuan Province between 1998 and 2001 and 61 PT1 strains collected in different regions in China between 1988 and 2005. The bacterial strain and plasmid screening results for spontaneous mutants of the *ompW* gene resistant to typing-phage VP5 are summarized in Table S1. The VP5 phage was propagated on host strain 2477c, as described in a previous study [23]. The phage titers were determined via a double-layer plaque assay. Unless otherwise stated, all strains were grown at 37 °C in Luria broth (LB) medium or on LB medium plates (15 g/L agar) supplemented with 1% NaCl. Antibiotics were used at the following concentrations: polymyxin B (PMB), 4 μg/mL; gentamicin (GM), 50 μg/mL.

### 2.2. DNA Extraction and Genome Sequencing

The Wizard Genomic DNA Extraction Kit (Promega, Madison, WI, USA) was used for genomic DNA extraction. Short-read sequencing was performed via the MGIEasy Universal DNA Library Prep set (Shenzhen, China) and the MGISEQ-200 platform (Shenzhen, China) following the manufacturer’s instructions. Oxford Nanopore Technologies (ONTs) platform (Oxford, UK) was used for long-read sequencing.

The qualities of the reads were checked via FastQC (Cambridge, UK). We then used Trimmomatic (v0.32) [24] software (Jülich, Germany) to trim low-quality ends. In addition, 50 bp was removed from the front and back ends of each clean data record. Short reads were assembled de novo into contigs and scaffolds using SPAdes (V3.7) (St. Petersburg, Russia; San Diego, CA, USA) [25]. To investigate the genomic organization within the prophage *V. cholerae* CTXΦ, nanopore long reads were assembled into a de novo draft genome using Miniasm (Houston, TX, USA) combined with Racon (Zagreb, Croatia). Assembly errors were corrected using Pilon version 1.23 (Cambridge, MA, USA) [26]. Assembly metrics were calculated using QUAST v4.5 (St. Petersburg, Russia; San Diego, CA, USA) [27]. The genomes were annotated and analyzed via Prokka v1.14.5 (Melbourne, Australia) [28] and the nr/nt database of NCBI (https://blast.ncbi.nlm.nih.gov/Blast.cgi, accessed on 17 May 2023), respectively [29]. Snippy v4.4.5 (Melbourne, Australia) was used to compare the assembled fasta file to the reference genome (accession AE003852/AE003853) and obtain the number of SNPs [9]. All the data were submitted to GenBank under BioProject ID no. PRJNA1173366.

### 2.3. Phylogenetic Analysis and Phylodynamic Assessment

In addition to the 70 PT6 and 61 PT1 genomes sequenced in this study, two PT6 strains sequenced in a previous study, and 370 representative reference sequences of the 7CP were included in the phylogenetic analysis. The accession numbers of the reference strain genomes are listed in Table S2. Phylogenetic analysis was performed via RAxML (randomized accelerated maximum likelihood) (Munich, Germany) alongside the GTR Gamma nucleotide model and the rapid hill algorithm with 1000 bootstraps [30]. TreeTime (Tübingen, Germany; Basel, Switzerland) was used to transform this ML tree topology into dated trees after the exclusion of outlier sequences for ancestral sequence reconstruction and evolutionary rates evaluation [31]. The straightforward migrations were additionally visualized as a circular plot utilizing TBtools v2.119 (Guangzhou, China) to convert tabular data into a graphical circular format [32].

### 2.4. Selection and Complementation of ompW Gene Mutants

A double-layer plaque assay was used to select the mutants. The detailed operation was as follows: 30 μL (ICDC-VC597) or 100 μL (ICDC-VC1488) of cell culture (10^8^ CFU/mL) was mixed with 10 mL of 50 °C of melted 0.7% LB agar and poured onto an LB agar plate, and when the upper layer solidified; 1 mL of VP5 (10^8^ PFU/mL) was dropped onto the plate and incubated overnight at 37 °C. Plaque formation indicated that the strain was sensitive to VP5; otherwise, some resistant mutant colonies appeared on the plate. Mutant colonies were picked and transferred to a fresh LB agar plate. They were verified again with a double-layer plaque assay. To determine mutation frequencies, the average number of mutants present in 1 mL of the culture was divided by the average total count of viable bacterial cells [33]. The experiments were repeated three times. Cultures of strain VC597 with and without VP5 were used as negative and positive controls, respectively.

The *ompW* gene of phage-resistant mutants was subsequently amplified via PCR using the primers ompW-T-F and ompW-T-R (Table S3). PCR was performed as follows: 95 °C for 3 min; 30 cycles at 94 °C for 40 s, 42.5 °C for 40 s, and 72 °C for 50 s; and 72 °C for 5 min. PCR products were visualized on 1% agarose gel, and target bands were subjected to Sanger sequencing with forward and reverse primers in both directions. Positive (VC597) and negative controls were also included.

To construct complemented mutants with an intact *ompW* gene, N16961 chromosomal DNA was used as the template for amplification via the primers pSRKGm-NdeI-ompW-F and pSRKGm-KpnI-ompW-R (Table S3). The PCR was performed as follows: 95 °C for 3 min; 30 cycles at 95 °C for 15 s, 64.5 °C for 15 s, and 72 °C for 50 s; and 72 °C for 5 min. The correctly sequenced and decontaminated PCR products were ligated with the purified pSRKGm plasmid digested with *Kpn*I/*Nde*I, generating the plasmid pSRKGm-ompW, which was conjugally transferred into the *ompW* gene mutant strains from the donor strain *E. coli* SM10 *λpir*. Transconjugants were selected on LB agar (PMB, 4 μg/mL; GM, 50 μg/mL). Colonies from the selected LB agar plates were amplified with the primers pSRKGm-ompW-2777F and pSRKGm-ompW-3914R after positive verification via a double-layer plaque assay. A final isopropyl-beta-D-thiogalactopyranoside (IPTG) concentration of 0.2 mM was used to initiate induction. The resulting transconjugants were subsequently confirmed via sequencing. As a negative control, empty vector plasmids were used.

## 3. Results

### 3.1. PT6 Strains in Sichuan Province That Emerged in 1998 and Disappeared by 2001

**Figure 1:**
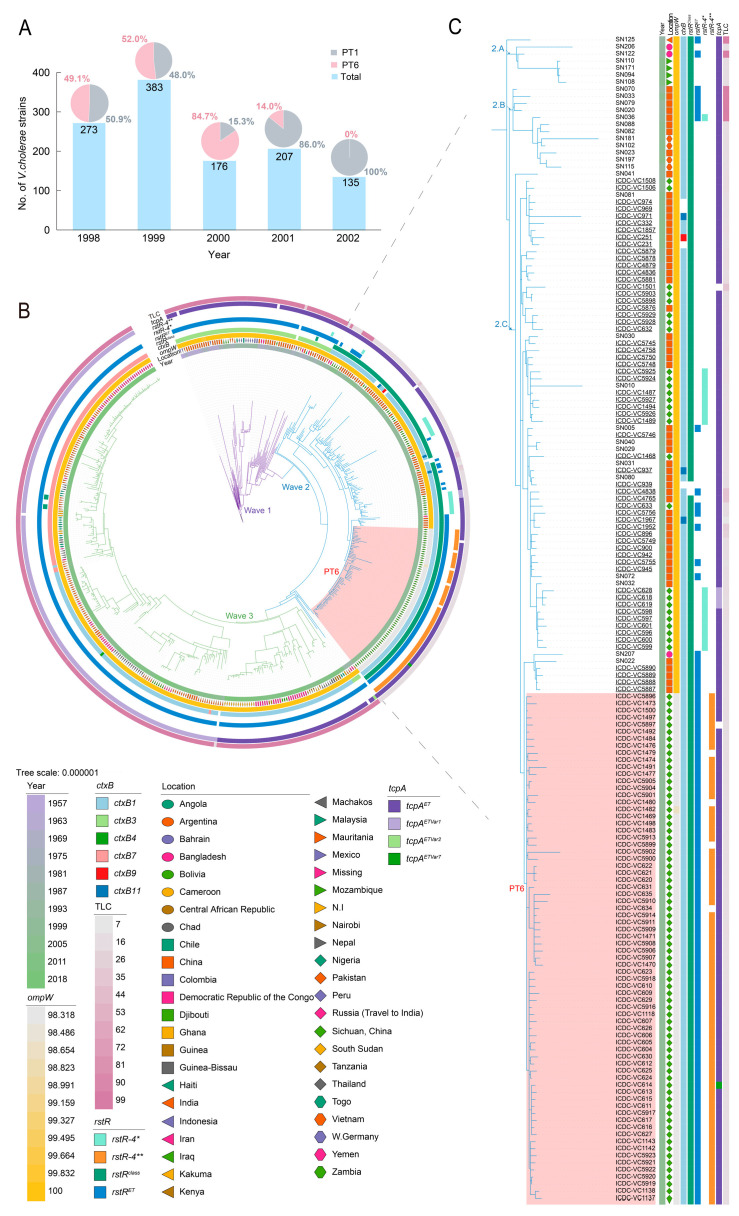
The molecular epidemiology of phage type 6 (PT6) and phage type 1 (PT1) strains in Sichuan Province. (**A**) *V. cholerae* strains collected in Sichuan Province from 1998 to 2002. (**B**) A maximum likelihood (ML) phylogenetic tree based on the single-nucleotide polymorphism (SNP) of 503 nonrepetitive, nonrecombinant core genomes of the El Tor *V. cholerae*. (**C**) The region in Figure 1B containing PT1 and PT6 strains with legible strain names enlarged for clarity. The PT1 and PT6 strains are highlighted with underlining and a pink background, respectively. The spatiotemporal information, the completeness of the *ompW* gene-coding sequence, the types of *ctxB* and *rstR* genes, and the presence of toxin-coregulated pilus A (*tcpA*) and toxin-linked cryptic (TLC) are also labeled from the inner to the outer circles. The isolation locations of PT6 strains were detailed at the county level. The scale bar represents the mean number of nucleotide substitutions per site. The phylogenetic tree with all strain names is illustrated in Figure S1.

A total of 1174 *V. cholerae* strains were observed during our surveillance from 1998 to 2002 in Sichuan Province, including 511 (43.5%, 511/1174) PT6 strains and 663 (56.5%, 663/1174) PT1 strains (Figure 1A). The PT6 strains exhibited a notable shift over time. These strains were initially identified in 1998 and are on par with PT1 strains. They then rapidly increased in prevalence, surpassing PT1 by 1999 and peaking in number. In 2000, nearly all strains were identified as PT6, the quantity of which was approximately six times greater than that of PT1. However, by 2001, a significant decrease was detected, with PT6 strains accounting for only 14.0% of the strains collected that year. Notably, the only strain identified in 2002 was PT1. PT6 strains have not been detected since that year throughout our epidemiological surveillance.

### 3.2. PT6 Strains Formed an Independent Clone Belonging to Wave 2

To clarify the evolutionary position of PT6 strains, a maximum likelihood (ML) phylogeny based on 3593 nonrepetitive, nonrecombinant core genome SNPs was obtained and rooted on A6 as a pre-7CP strain (Figure 1B, Figure S1) [34]. All PT6 strains formed an independent clone belonging to the 2.C clade in wave 2 of the 7CP, according to a previous study [14], suggesting that the Sichuan cholera epidemic was attributable to the single clonal dissemination of the 7CP strains (Figure 1C). Most PT6 strains presented the same 11 bp deletion in the *ompW* gene, with the exception of VC1482, which presented a deletion of 9 bp at nucleotide positions 298–306 and an “A” to “G” transition at nucleotide 308. In addition, the PT1 strains sequenced in this study were nested in clade 2.C, which indicated that they shared a common origin with PT6. In addition, among the strains exhibiting the closest genetic relationship with the PT6 group, all strains from China were isolated in 2001, with the exception of SN207, which was isolated from Bangladesh in 2000. These strains possessed an intact *ompW* gene in contrast to PT6, suggesting that the PT6 clone might have originated from a strain with an intact *ompW* gene.

In addition, we investigated the features of prominent virulence elements of *V. cholerae*, including the genes *ctxB*, *rstR*, and *tcpA*, as well as toxin-linked cryptic (TLC) gene clusters, which are genetic elements related to the acquisition or replication of CTXΦ (Figure 1C). All PT6 strains contained the *ctxB1* gene. Notably, the *rstR* gene presented distinct alleles, including *rstR^class^*, *rstR^ET^*, and rstR-4**. Despite the first two genes being present in all PT6 strains, rstR-4** was absent in VC634, VC1479, VC1480, and VC5899. Interestingly, the rstR-4** gene was initially identified in the sequence of the O27 serogroup *V. cholerae* strain SCE223 (AF133307), which was obtained from water samples in Calcutta, India [35]. In addition, the rstR-4** gene in the PT6 strains encoded the same protein as rstR-4** from SCE223, but a single-nucleotide substitution, T237C, emerged. Furthermore, all PT6 strains harbored the *tcpA^ET^* gene in addition to the deletion and mutation in the strains VC5897 and VC614, respectively. Variants of the *tcpA^ETVar7^* gene were differentiated from *tcpA^ET^* in the variable nucleotide positions of T11C, A259G, and C428T. Additionally, all PT6 strains eliminated the TLC element. Like PT6, all PT1 strains harbored *ctxB1* and *rstR^class^*, except for VC4838, which lacked the latter. Among them, 12 PT1 strains possessed the *rstR^ET^* allele, and 16 strains carried the rstR-4* allele, which was identified from an environmental isolate that contained two overlapping open reading frames in comparison to rstR-4** [35]. Analogous to the PT6 strains, nearly all PT1 strains lacked the TLC, with the exception of a few strains that possessed an incomplete TLC. Notably, three PT1 strains (VC618/619/628) harbored the *tcpA^ETVar1^* (A266G) variant, whereas VC1501 was devoid of *tcpA*.

### 3.3. Multiple Copies of RS1^ET^ or RS1^ET^ with RS1-4** Integrated on Chr. 1 of PT6 Strains

**Figure 2:**
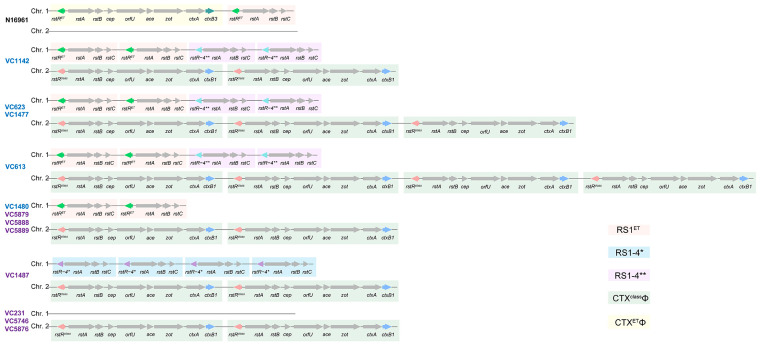
Representative organization of the CTXΦ prophage and its satellite in *V. cholerae* strains. The PT6 and PT1 strains are marked in dark blue and dark purple, respectively. Differently colored boxes are used to distinguish various types of CTXΦ and RS1. Genes, including *rstR* and *ctxB*, involved in identifying the CTXΦ and RS1 types are distinguished using different colors.

To understand the structure of the CTXΦ and RS1 genomic regions, we further constructed a complete map of the *V. cholerae* genomes from five PT6 strains, seven PT1 representative strains, and the N16961 reference strain (Figure 2). The PT6 strains presented between two and four copies of CTX^class^Φ on Chr. 2 and either two tandemly arrayed copies of RS1^ET^ or a combination of two copies of RS1^ET^ and two copies of RS1-4** on Chr. 1. In contrast, CTX^ET^Φ and RS1^ET^ were exclusively integrated on Chr. 1 in the N16961 strain. In addition, three PT1 strains, including two strains (VC5888 and VC5889) that shared the closest evolutionary relationships with the PT6 group and strain VC5879, exhibited similar integration patterns to those of the PT6 strain (VC1480). In addition, two additional arrays of CTXΦ and RS1 were present in the PT1 strains. Both strains carried two tandem arrayed copies of CTX^class^Φ on Chr. 2, whereas VC1487 also harbored four copies of RS1-4* on Chr. 1. Overall, our findings suggest that PT6 strains retained the key characteristics of the wave 2 pandemic strains, exhibiting a tandem repeat of CTX^class^Φ on Chr. 2, while they also carried RS1-4**, suggesting unique evolutionary traits for this clone.

### 3.4. Frequent Cross-Regional Transmission and Local Outbreaks in Sichuan

To examine the transmission pathways of the PT6 strains in greater detail, we inferred the ancestral geographical locations of branches via time-stamped phylogeny. The most recent common ancestor (MRCA) of the PT6 strains was estimated to be from August 1991 (95% high posterior density [HPD], April 1991 to June 1992) (Figure S2). The estimated mutation rate was 5.0 ± 2.1 with a standard deviation (SD) of ×10^−7^ SNPs/site/year, which is approximately twice the rate of 7CP strains [9].

**Figure 3:**
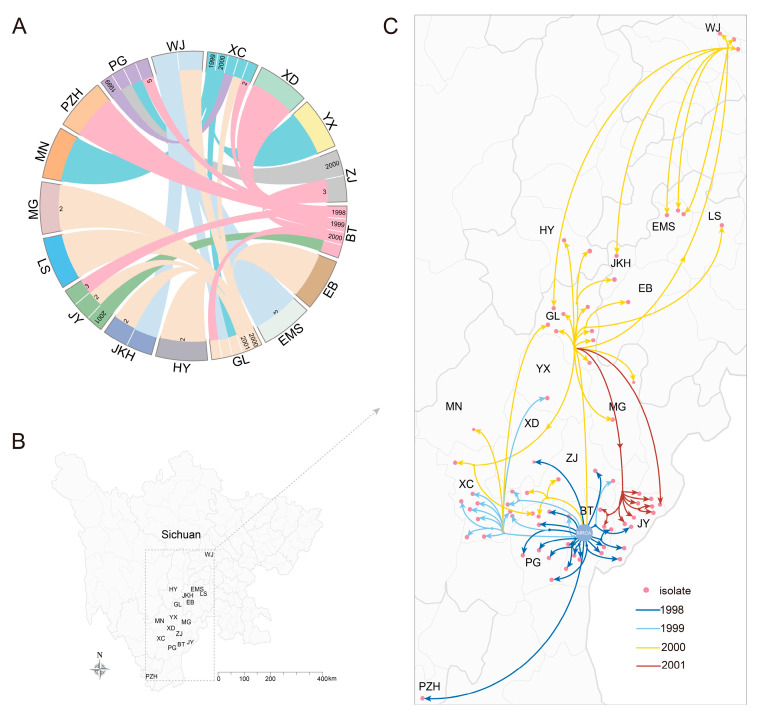
The inferred migrations of PT6 strains between geographical countries and local transmission locations in Sichuan Province. (**A**) A circular plot depicting the inferred cross-district migratory patterns of PT6 strains in Sichuan Province. The flow bars share the same color as the sources of transmissions. One end of each bar directly touches the location of the origin, whereas the other end terminates at the location of the destination and marks the number of introductions if it exceeds one occasion. A particular year for each migration event is indicated at the original end of the respective bars. (**B**) A map of Sichuan Province. An abbreviated designation of the region where PT6 strains were isolated is shown on the map. (**C**) The detailed transmission routes of the epidemic PT6 strains in Sichuan. Abbreviations: BT, Butuo County, Liangshan Prefecture; EB, Ebian County, Leshan City; EMS, Emeishan City, Leshan City; GL, Ganluo County, Liangshan Prefecture; HY, Hanyuan County, Ya’ an City; JKH, Jinkouhe District, Leshan City; JY, Jinyang County, Liangshan Prefecture; LS, Leshan City central region; MG, Meigu County, Liangshan Prefecture; MN, Mianning County, Liangshan Prefecture; MRCA, the most recent common ancestor; PG, Puge County, Liangshan Prefecture; PZH, Panzhihua City central region; WJ, Wenjiang District, Chengdu City; XC, Xichang City, Liangshan Prefecture; XD, Xide County, Liangshan Prefecture; YX, Yuexi County, Liangshan Prefecture; ZJ, Zhaojue County, Liangshan Prefecture.

We then used a circular plot to illustrate the cross-regional transmission of the PT6 strains, identifying forty events involving several counties in Liangshan Prefecture, Chengdu (CD) City, Leshan (LS) City, Panzhihua (PZH) City and Ya’an (YA) City (Figure 3A, Table S4). Our data suggest that the PT6 clone initially appeared in Butuo (BT) County, which is located in the southern Liangshan Prefecture, Sichuan Province (Figure 3B and Figure S2). Between 1998 and 2000, a total of 16 BT cases were disseminated into other regions. Strains from BT initially disseminated to nearby regions, such as Jinyang (JY), Puge (PG), Zhaojue (ZJ), Xide (XD), and Xichang (XC), with notable cross-city transmissions to PZH. However, in 2000, strains transmitted from BT to Ganluo (GL), the northernmost county of Liangshan, and triggered an outbreak that spread northwards (Figure 3B, C). Multiple spillover events from GL to outside Liangshan Prefecture, including those reaching LS (e.g., Ebian [EB] County, Jinkouhe [JKH] County, and the urban area), YA (Hanyuan [HY] County), and CD (Wenjiang [WJ] District), occurred (Figure 3C). In fact, the cross-regional transmission events caused by BT and GL as transmission sources accounted for more than half of the total transmission events (28/40, 70.0%), indicating that BT and GL play crucial roles as important hubs in cholera prevention and control. Despite the widespread dissemination of PT6 strains in 2000, they began to spread southwards in 2001, remaining confined to Liangshan Prefecture, such as strains from GL to JY, before returning to BT (Figure 3C). Moreover, severe local prevalence and extension were also identified; examples include the local outbreak in BT in 1998 and the subsequent local prevalence and dissemination following cross-regional transmission, such as in XC in 1999, GL in 2000, and JY in 2001 (Figure 3C). In brief, the PT6 strains demonstrated a pattern of dissemination from south to north within Sichuan Province, subsequently returning south to its original emergence location (BT) before vanishing, providing valuable insights into the migratory patterns of *V. cholerae* in question.

### 3.5. Spontaneous Mutations of the ompW Gene in V. cholerae Under VP5 Phage Pressure

A signature mutation comprising an 11 bp deletion in *ompW* occurred in the PT6 strains [18]. We then estimated the spontaneous mutation frequency of the *ompW* gene under VP5 phage infection pressure, which may promote an understanding of the emergence of PT6 strains possessing VP5 resistance. The PT1 wild-type strains VC597 and VC1488, which share the intact and identical coding regions of the *ompW* gene (654 bp) with the strain N16961, were screened for spontaneous mutations of the *ompW* gene under pressure from typing phage VP5. A total of 56 mutants were obtained by sequencing the *ompW* gene (Table 1). The frequencies of spontaneous mutations of the *ompW* gene, resulting in the resistance of VC597 and VC1488 towards typing phage VP5, were 9.9 × 10^−6^ CFU/mL and 5.6 × 10^−6^ CFU/mL, respectively. Nucleic acid variations, such as deletions, insertions, and substitutions, were included. Deletions (66.1%, 37/56) were the most common type of mutation, followed by insertions (28.6%, 16/56) and substitutions (5.3%, 3/56). The deletion mutation predominantly occurred in the region spanning 0 to 100 bp (40.5%, 15/37) in *ompW*, whereas the insertion mutation primarily occurred between 200 and 300 bp (68.8%, 11/16). Substitution mutations were found only in 494 nt (33.3%, 1/3) and 542 nt (66.7%, 2/3), especially dual mutations (insertion and substitution), which appeared at the latter position. Therefore, the *ompW* gene in *V. cholerae* is prone to diverse types of nucleotide mutations at different loci within the gene.

## 4. Discussion

In our previous cholera surveillance, we discovered a distinct class of phage-type strains (PT6) via the phage biotyping scheme, which triggered a four-year epidemic in certain regions of Sichuan [18]. These strains are distinguished by an 11 bp deletion in the *ompW* gene, resulting in resistance to infection by typing phage VP5. Consequently, in contrast to a prior study that narrowly focused on the singular phenotype of phage susceptibility in PT6 strains and their *ompW* mutation, here, we provide a comprehensive genomic re-evaluation of these isolates. We demonstrate that PT6 strains constitute an independent clone exhibiting diversified branches. Critically, the phylogenomic analysis revealed clear segregation from co-circulating PT1 strains. Through high-resolution complete genome assemblies, we further identified conserved CTXΦ and RS1 prophage architectures mirroring Wave2 isolates [36], while elevated CTXΦ copy numbers in later epidemic PT6 strains correlated with enhanced toxigenicity. In addition, the integration of spatiotemporal metadata with genomic variations enabled the high-precision reconstruction of PT6 transmission networks across Sichuan Province, surpassing traditional spatiotemporal modeling. Notably, we provide experimental evidence that the *ompW* gene undergoes recurrent spontaneous mutations under VP5 phage selection pressure, yielding multiple mutant variants.

In fact, the methodology of genomic epidemiology employed in this study holds promise for broader applications within public health. The whole-genome sequencing of pathogens not only enables the tracking of transmission dynamics and the identification of sources and transmission routes of infectious diseases but also facilitates the discovery of previously unrecognized pathogens [37]. This capability is crucial for formulating more effective control strategies and targeted intervention measures and enabling rapid responses to cross-regional outbreaks. Furthermore, genomic epidemiology can be effectively integrated into routine surveillance and the control of infectious diseases [38]. When combined with traditional epidemiological approaches, this powerful tool provides deeper insights into the origins, transmission pathways, and extent of pathogen spread. Additionally, the identification and investigation of mutations in microbial receptors are of significant importance in fundamental biology, as such mutations can profoundly impact microbial viability, adaptability, and pathogenicity [39,40]. This study revealed that the *ompW* gene of *V. cholerae* can undergo diverse mutations under bacteriophage VP5 pressure. We hypothesize that the emergence of these mutations is likely linked to adaptive responses to environmental stressors. The in-depth investigation of these mutations and their effects on bacterial fitness will provide critical insights for developing novel antimicrobial strategies.

**Figure 4:**
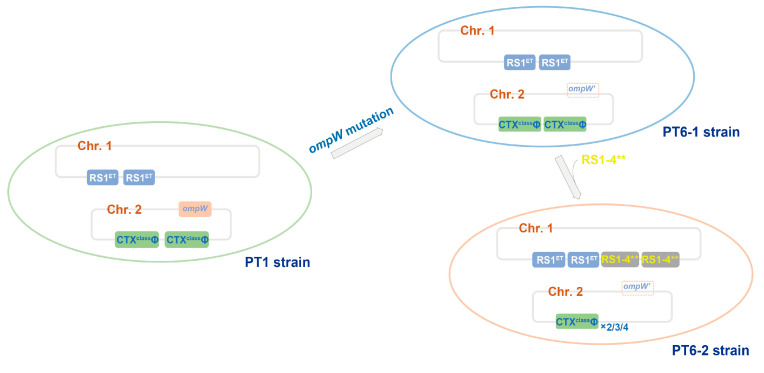
A schematic illustration of the speculated generation of CTXΦ and RS1 elements in the PT6 strains. *ompW’* denotes the mutant *ompW* gene.

The origin of the PT6 strains is also a matter of interest in our research process. Phylogenetic analysis of the core genomes revealed that both the PT1 and PT6 strains belonged to clade 2.C, with their most recent common ancestor being genetically closest to the PT1 strain SN041. In addition, compared to some PT1 strains, four PT6 strains (VC634/1479/1480/5899) without RS1-4** (PT6-1) detected in the early years (1998 and 1999) merely presented an 11 bp deletion mutation in *ompW* in the core genome. Nevertheless, all strains (SN207, SN022, ICDC-VC5890/5889/5888/5887) phylogenetically nearest the PT6 strains possessed intact *ompW*, suggesting that they shared a common ancestor harboring intact *ompW* with PT6. Hence, we speculated that PT6 strains, which emerged over a period of four years, originated from a prototype strain of PT1 following *ompW* mutation (Figure 4). In the majority of PT6 strains (PT6-2), there were also two copies of RS1-4** on Chr. 1, indicating that the introduction of RS1-4** likely occurred subsequently to the *ompW* mutation. Consequently, we inferred that the PT1 strains initially underwent *ompW* mutation, which subsequently led to the formation of the PT6 strains after acquiring RS1-4** in Sichuan. Alternatively, it is plausible that PT6-1 and PT6-2 were concurrently introduced into Sichuan, leading to an epidemic outbreak. Among these, PT6-1 did not establish dominance, whereas PT6-2 emerged as the predominant variant.

Both PT6-1 and PT6-2 were confined to circulation in Sichuan, especially in Liangshan. This restriction is speculated to be due not only to the relatively inferior economic development and sanitation conditions that exist compared to those in other regions of Sichuan but also the local customs surrounding luxurious funeral banquets, which led to the rapid spread of the strain in the area [41]. According to existing research, sociocultural determinants play a significant role in facilitating the transmission of cholera between individuals [42]. Therefore, when the strain dispersed from Liangshan Prefecture to other regions, improvements in sanitary conditions, shifts in social customs, and the consequentially enhanced surveillance schemes in response to the outbreak curtailed its ongoing transmission.

For PT6 strains with propagation space–time propagation constraints, we employed genomic phylogenetic analysis in conjunction with epidemiological data, such as the temporal and geographical occurrence of bacterial infection cases, to construct a diffusion route map of PT6 strains. This elucidated the transmission pathways of *V. cholerae* across various temporal and spatial contexts. Conducting phylogenetic analysis in real time throughout the transmission process can elucidate the transmission pathway, trace the source of infection and its spread, enable precise guidance, control the origin of transmission, and prevent the propagation of further infections. Genomics and phylogenetic studies have been listed as the core components of the World Health Organization (WHO) global framework to define and guide studies into the origins of emerging and re-emerging pathogens with epidemic and pandemic potential, which further underscores the imperative of executing genomic epidemiological analyses to comprehend the dissemination of outbreaks and enact effective control measures [43].

The potential emergence of phage-resistant mutants challenges the utilization of phages for the treatment of infectious diseases [44]. Among the mechanisms of antiphage-like responses, mutations impacting phage receptors are a crucial factor contributing to phage resistance [45]. OmpW, as the receptor for VP5, plays a key role in bacterial survival and pathogenicity [46]. Our study revealed that under the selective pressure exerted by typing phage VP5, wild-type *V. cholerae* can experience spontaneous mutations in the *ompW* gene, resulting in the emergence of mutant strains capable of resisting VP5 and thus surviving. Notably, the *ompW* of *V. cholerae* presented a variety of mutation types in our study, with mutation rates of the order of 10^−6^ CFU/mL. The frequency of *Yersinia pestis* spontaneous mutations associated with bacteriophage resistance was in the range of 10^−4^ CFU/mL to 10^−7^ CFU/mL, and some were even below 10^−10^ CFU/mL [33], suggesting that OmpW, an outer membrane component susceptible to mutations, plays a notable role in facilitating the survival of the strain by conferring resistance to phage infection.

In this study, we used genome sequencing and phylogenetic analysis to examine the variation and transmission dynamics of the PT6 clone. Our analysis establishes a research framework and provides insights into transmission patterns for understanding the spread of cholera, particularly during localized outbreaks. This offers actionable guidance for public health policymakers, enabling them to more accurately trace the origins and transmission routes of cholera outbreaks, thereby facilitating more effective resource allocation and targeted interventions. Crucially, the genomic epidemiology approach employed in this study provides significantly richer epidemiological intelligence compared to traditional diagnostic methods—such as phage lysis assays—which typically yield only singular data points. This comparative advantage also offers valuable references for countries or regions still reliant on conventional diagnostic approaches to cholera surveillance, particularly in resource-limited settings. As antimicrobial resistance continues to increase [47], our findings highlight the risks of relying on monophage therapy and the ability to optimize phage selection to prevent resistance and enhance our understanding of phage–bacteria interactions. Although there have been no reports of PT6 strains since 2001, research into the genomic characteristics of the early epidemic strains associated with the 7CP may provide valuable insights for the future prevention and control of cholera [16]. As a result, our study enhances the understanding of *V. cholerae* biology by providing a detailed characterization of a specific phage-typing clone with unique genetic markers and epidemiological and genomic features.

## Figures and Tables

**Table 1:** Base differences between mutants with N16961 in the *ompW* gene of *V. cholerae*.

Strain	No. of Mutants	Mutation Rate	Mutation Type		
			Deletion (66.1%, 37/56)	Insertion (28.6%, 16/56)	Substitution (5.3%, 3/56)
VC597	19	9.9 × 10^−6^ CFU/mL	79–90 nt (15.8%, 3/19); 80–90 nt (31.6%, 6/19); 89–90 nt (5.3%, 1/19); 167 nt (5.3%, 1/19); 322–323 nt (5.3%, 1/19); 520–523 nt (10.5%, 2/19); 545–586 nt (5.3%, 1/19)	288 nt: TG (5.3%, 1/19); 374 nt: ATG (5.3%, 1/19)	G542A (10.5%, 2/19)
VC1488	37	5.6 × 10^−6^ CFU/mL	15–31 nt (2.7%, 1/37); 84–85 nt (10.8%, 4/37); 326–336 nt (2.7%, 1/37); 327–328 nt (13.5%, 5/37); 365–371 nt (16.2%, 6/37); 422–431 nt (2.7%, 1/37)	235 nt: G (2.7%, 1/37); 263 nt: C (2.7%, 1/37); 291 nt: T (21.6%, 8/37); 479 nt: T (5.4%, 2/37); 538 nt: G (2.7%, 1/37); 542 nt: G (2.7%, 1/37)	G494A (2.7%, 1/37)
			502–512 nt (2.7%, 1/37); 537–538 nt (2.7%, 1/37); 608–609 nt (2.7%, 1/37); 635–639 nt (2.7%, 1/37)		

## Data Availability

The original contributions presented in this study are included in the article/Supplementary Material. Further inquiries can be directed to the corresponding authors.

## Supplementary Materials

The following supporting information can be downloaded at https://www.mdpi.com/article/10.3390/microorganisms13071585/s1. Figure S1: The nonrepetitive, nonrecombinant core genome SNP-based phylogenomic tree using ML of 503 El Tor *V. cholerae* strains; Figure S2: Time-calibrated whole-genome phylogeny of 72 PT6 strains in addition to 402 reference genomes sampled worldwide. PT6 strains from various years are marked with different background colors; Table S1: Strains and plasmids used in this study; Table S2: The background information of strains identified in this study and reference strains; Table S3: Oligonucleotide primers used in this study; Table S4: Inferred migrations of PT6 strains between geographical locations in Sichuan Province.
