# Supplementary material for: Revisiting the Genomic Epidemiology of Distinct Phage-Type Vibrio cholerae Strains Reveals Restricted Spatiotemporal Dissemination During an Epidemic
Source: Microorganisms. 2025 Jul 5;13(7):1585. doi: 10.3390/microorganisms13071585 (PMC12300545; doi:10.3390/microorganisms13071585)
Supplement: Supplementary file 1 [file microorganisms-13-01585-s001.zip › Supplementary Table S1-S4.pdf]

# Revisiting the Genomic Epidemiology of Distinct Phage-Type *Vibrio cholerae* Strains Reveals Restricted Spatiotemporal Dissemination During an Epidemic

**Table S1. Strains and plasmids used in this study.**

| Strains or plasmids | Relevant genotype | Resource |
|---|---|---|
| *E. coli* | | |
| SM10 λpir | Km$^r$ *thi-1 thr leu tonA lacY supE recA::RP4-2-Tc::Mu λpir* | Laboratory stock |
| *V. cholerae* | | |
| 2477c | O1 El Tor, Ogawa | Laboratory stock |
| N16961 | O1 El Tor, Inaba, Sm$^r$ | Laboratory stock |
| ICDC-VC597 | Wild-type El Tor VC597, VP5 sensitive, abbreviated as VC597 | Laboratory stock |
| ICDC-VC1488 | Wild-type El Tor VC1488, VP5 sensitive, abbreviated as VC1488 | Laboratory stock |
| Plasmids | | |
| pSRKGm | Broad host range expression vector with tightly regulated, IPTG inducible lacZ promoter; Gm$^r$ | Laboratory stock |
| pSRKGm-ompW | pSRKGm-derived, N16961 (ompW), Gm$^r$ | This study |

**Table S2. The background information of strains identified in this study and reference strains.**

| Serial Number | Year | Location | Sources |
|---|---|---|---|
| ICDC-VC1118 | 2000 | Ganluo, China | This study |
| ICDC-VC1137 | 2001 | Butuo, China | This study |
| ICDC-VC1138 | 2001 | Butuo, China | This study |
| ICDC-VC1142 | 2001 | Jinyang, China | This study |
| ICDC-VC1143 | 2001 | Jinyang, China | This study |
| ICDC-VC1469 | 1999 | Xichang, China | This study |
| ICDC-VC1470 | 1999 | Xichang, China | This study |
| ICDC-VC1471 | 1999 | Xichang, China | This study |
| ICDC-VC1473 | 1998 | Xide, China | This study |
| ICDC-VC1474 | 1998 | Jinyang, China | This study |
| ICDC-VC1476 | 1998 | Jinyang, China | This study |
| ICDC-VC1477 | 1998 | Butuo, China | This study |
| ICDC-VC1480 | 1998 | Butuo, China | This study |
| ICDC-VC1482 | 1998 | Puge, China | This study |
| ICDC-VC1483 | 1998 | Puge, China | This study |
| ICDC-VC1484 | 1998 | Puge, China | This study |

| | | | |
|---|---|---|---|
| ICDC-VC1491 | 1998 | Panzhihua, China | This study |
| ICDC-VC1492 | 1998 | Puge, China | This study |
| ICDC-VC1497 | 1998 | Butuo, China | This study |
| ICDC-VC1498 | 1998 | Butuo, China | This study |
| ICDC-VC1500 | 1998 | missing, China | This study |
| ICDC-VC5896 | 1998 | Butuo, China | This study |
| ICDC-VC5897 | 1998 | Butuo, China | This study |
| ICDC-VC5899 | 1998 | Jinyang, China | This study |
| ICDC-VC5900 | 1998 | Puge, China | This study |
| ICDC-VC5901 | 1998 | Puge, China | This study |
| ICDC-VC5902 | 1998 | Zhaojue, China | This study |
| ICDC-VC5904 | 1999 | Xichang, China | This study |
| ICDC-VC5905 | 1999 | Xichang, China | This study |
| ICDC-VC5906 | 1999 | Xichang, China | This study |
| ICDC-VC5907 | 1999 | Xichang, China | This study |
| ICDC-VC5908 | 1999 | Xichang, China | This study |
| ICDC-VC5909 | 1999 | Xichang, China | This study |
| ICDC-VC5910 | 1999 | Xichang, China | This study |
| ICDC-VC5911 | 1999 | Xichang, China | This study |
| ICDC-VC5913 | 1999 | Zhaojue, China | This study |
| ICDC-VC5914 | 2000 | Ganluo, China | This study |
| ICDC-VC5916 | 2000 | Ganluo, China | This study |
| ICDC-VC5917 | 2000 | Ganluo, China | This study |
| ICDC-VC5918 | 2001 | Jinyang, China | This study |
| ICDC-VC5919 | 2001 | Jinyang, China | This study |
| ICDC-VC5920 | 2001 | Jinyang, China | This study |
| ICDC-VC5921 | 2001 | Jinyang, China | This study |
| ICDC-VC5922 | 2001 | Jinyang, China | This study |
| ICDC-VC5923 | 2001 | Jinyang, China | This study |
| ICDC-VC604 | 2000 | Ebian, China | This study |
| ICDC-VC606 | 2000 | Ganluo, China | This study |
| ICDC-VC607 | 2000 | Ganluo, China | This study |
| ICDC-VC609 | 2000 | Ganluo, China | This study |
| ICDC-VC610 | 2000 | Ganluo, China | This study |
| ICDC-VC611 | 2000 | Jinkouhe, China | This study |
| ICDC-VC612 | 2000 | Jinkouhe, China | This study |
| ICDC-VC613 | 2000 | Emeishan, China | This study |
| ICDC-VC614 | 2000 | Emeishan, China | This study |
| ICDC-VC615 | 2000 | Emeishan, China | This study |
| ICDC-VC616 | 2000 | Wenjiang, China | This study |
| ICDC-VC617 | 2000 | Wenjiang, China | This study |
| ICDC-VC620 | 2000 | Zhaojue, China | This study |
| ICDC-VC621 | 2000 | Zhaojue, China | This study |
| ICDC-VC622 | 2000 | Puge, China | This study |
| ICDC-VC623 | 2000 | Meigu, China | This study |
| ICDC-VC624 | 2000 | Puge, China | This study |
| ICDC-VC625 | 2000 | Xichang, China | This study |
| ICDC-VC626 | 2000 | Meigu, China | This study |

| | | | |
|---|---|---|---|
| ICDC-VC627 | 2000 | Wenjiang, China | This study |
| ICDC-VC629 | 2000 | Hanyuan, China | This study |
| ICDC-VC630 | 2000 | Hanyuan, China | This study |
| ICDC-VC631 | 1999 | Butuo, China | This study |
| ICDC-VC634 | 1999 | Mianning, China | This study |
| ICDC-VC635 | 1999 | Yuexi, China | This study |
| ICDC-VC1468 | 1999 | China | This study |
| ICDC-VC1487 | 1998 | China | This study |
| ICDC-VC1489 | 1998 | China | This study |
| ICDC-VC1494 | 1998 | China | This study |
| ICDC-VC1952 | 2000 | China | This study |
| ICDC-VC1967 | 2000 | China | This study |
| ICDC-VC4758 | 1997 | China | This study |
| ICDC-VC4765 | 1998 | China | This study |
| ICDC-VC4836 | 1999 | China | This study |
| ICDC-VC4838 | 1999 | China | This study |
| ICDC-VC5745 | 1997 | China | This study |
| ICDC-VC5746 | 1997 | China | This study |
| ICDC-VC5748 | 1998 | China | This study |
| ICDC-VC5749 | 1998 | China | This study |
| ICDC-VC5750 | 1998 | China | This study |
| ICDC-VC5756 | 2000 | China | This study |
| ICDC-VC5898 | 1998 | China | This study |
| ICDC-VC5903 | 1998 | China | This study |
| ICDC-VC5924 | 1998 | China | This study |
| ICDC-VC5925 | 1998 | China | This study |
| ICDC-VC5926 | 1998 | China | This study |
| ICDC-VC5927 | 1998 | China | This study |
| ICDC-VC5928 | 1999 | China | This study |
| ICDC-VC5929 | 1999 | China | This study |
| ICDC-VC596 | 2000 | China | This study |
| ICDC-VC597 | 2000 | China | This study |
| ICDC-VC598 | 2000 | China | This study |
| ICDC-VC599 | 2000 | China | This study |
| ICDC-VC600 | 2000 | China | This study |
| ICDC-VC601 | 2000 | China | This study |
| ICDC-VC618 | 2000 | China | This study |
| ICDC-VC619 | 2000 | China | This study |
| ICDC-VC628 | 2000 | China | This study |
| ICDC-VC632 | 1999 | China | This study |
| ICDC-VC633 | 1999 | China | This study |
| ICDC-VC1501 | 1997 | China | This study |
| ICDC-VC1506 | 1997 | China | This study |
| ICDC-VC1508 | 1997 | China | This study |
| ICDC-VC1857 | 2004 | China | This study |
| ICDC-VC231 | 2004 | China | This study |
| ICDC-VC251 | 2004 | China | This study |
| ICDC-VC332 | 2005 | China | This study |

| | | | |
|---|---|---|---|
| ICDC-VC4879 | 1988 | China | This study |
| ICDC-VC5755 | 1999 | China | This study |
| ICDC-VC5876 | 1998 | China | This study |
| ICDC-VC5878 | 1998 | China | This study |
| ICDC-VC5879 | 1998 | China | This study |
| ICDC-VC5881 | 1999 | China | This study |
| ICDC-VC5887 | 2001 | China | This study |
| ICDC-VC5888 | 2001 | China | This study |
| ICDC-VC5889 | 2001 | China | This study |
| ICDC-VC5890 | 2001 | China | This study |
| ICDC-VC896 | 1998 | China | This study |
| ICDC-VC900 | 2001 | China | This study |
| ICDC-VC937 | 1998 | China | This study |
| ICDC-VC939 | 1998 | China | This study |
| ICDC-VC942 | 1999 | China | This study |
| ICDC-VC945 | 1999 | China | This study |
| ICDC-VC969 | 2002 | China | This study |
| ICDC-VC971 | 2002 | China | This study |
| ICDC-VC974 | 2002 | China | This study |
| ICDC-VC1479 | 1998 | Butuo, China | ERP006433 |
| ICDC-VC605 | 2000 | Leshan, China | ERP006434 |
| SN004 | 2005 | China | CP009041/CP009042 |
| SN005 | 1998 | China | ERP006431 |
| SN010 | 1998 | China | ERP006432 |
| SN013 | 2008 | China | ERP006435 |
| SN014 | 2005 | China | ERP006436 |
| SN019 | 1984 | China | ERP006437 |
| SN020 | 1994 | China | ERP006438 |
| SN021 | 1978 | China | ERP006439 |
| SN022 | 2001 | China | ERP006440 |
| SN023 | 2002 | China | ERP006441 |
| SN025 | 1979 | China | ERP006443 |
| SN026 | 1990 | China | ERP006444 |
| SN027 | 1991 | China | ERP006445 |
| SN028 | 1992 | China | ERP006446 |
| SN029 | 1993 | China | ERP006447 |
| SN030 | 1997 | China | ERP006448 |
| SN031 | 1998 | China | ERP006449 |
| SN032 | 2000 | China | ERP006450 |
| SN033 | 2001 | China | ERP006452 |
| SN034 | 2008 | China | ERP006453 |
| SN035 | 1978 | China | ERP006454 |
| SN036 | 1996 | China | ERP006455 |
| SN037 | 1980 | China | ERP006457 |
| SN038 | 1985 | China | ERP006458 |
| SN040 | 1998 | China | ERP006460 |
| SN041 | 1995 | China | ERP006461 |
| SN042 | 1986 | China | ERP006462 |

| | | | |
|---|---|---|---|
| SN044 | 1981 | China | ERP006464 |
| SN045 | 1987 | China | ERP006465 |
| SN046 | 1964 | China | ERP006466 |
| SN047 | 1979 | China | ERP006467 |
| SN048 | 1988 | China | ERP006468 |
| SN049 | 1961 | China | ERP006469 |
| SN050 | 1961 | China | ERP006470 |
| SN053 | 1966 | China | ERP006473 |
| SN054 | 1962 | China | ERP006474 |
| SN055 | 1964 | China | ERP006475 |
| SN056 | 1965 | China | ERP006476 |
| SN057 | 1969 | China | ERP006477 |
| SN058 | 1973 | China | ERP006478 |
| SN059 | 1974 | China | ERP006479 |
| SN060 | 1977 | China | ERP006480 |
| SN061 | 1978 | China | ERP006481 |
| SN062 | 1979 | China | ERP006482 |
| SN063 | 1980 | China | ERP006483 |
| SN064 | 1981 | China | ERP006484 |
| SN065 | 1982 | China | ERP006485 |
| SN066 | 1983 | China | ERP006486 |
| SN067 | 1983 | China | ERP006487 |
| SN068 | 1984 | China | ERP006488 |
| SN069 | 1988 | China | ERP006489 |
| SN070 | 1993 | China | ERP006490 |
| SN072 | 1999 | China | ERP006492 |
| SN073 | 1961 | Indonesia | ERP006493 |
| SN074 | 1961 | Indonesia | ERP006494 |
| SN076 | 1963 | China | ERP006496 |
| SN077 | 1964 | China | ERP006497 |
| SN078 | 1986 | Mauritania | ERP006498 |
| SN079 | 1993 | China | ERP006499 |
| SN080 | 1998 | China | ERP006500 |
| SN081 | 2001 | China | ERP006501 |
| SN082 | 2001 | China | ERP006502 |
| SN083 | 2010 | China | ERP006503 |
| SN084 | 1991 | Peru | ERP006504 |
| SN086 | 1961 | China | ERP006506 |
| SN088 | 2001 | China | ERP006508 |
| SN089 | 1991 | Chile | ERP006509 |
| SN091 | 1991 | Peru | ERP006510 |
| SN094 | 2005 | Mozambique | ERS013265 |
| SN095 | 1991 | Bangladesh | ERS013282 |
| SN096 | 1989 | India | ERS013130 |
| SN098 | 2007 | India | ERS013233 |
| SN099 | 1977 | India | ERS013248 |
| SN101 | 2007 | India | ERS013232 |
| SN102 | 2003 | Vietnam | ERS013273 |

| | | | |
|---|---|---|---|
| SN103 | 2007 | Nairobi | ERS013213 |
| SN104 | 2009 | Machakos | ERS013226 |
| SN105 | 1971 | Bangladesh | ERS013249 |
| SN106 | 2004 | India | ERS013254 |
| SN107 | 2006 | Bangladesh | ERS013129 |
| SN108 | 2005 | Mozambique | ERS013242 |
| SN110 | 2004 | Mozambique | ACHZ00000000 |
| SN112 | 1991 | Bangladesh | ERS013135 |
| SN114 | 1991 | Peru | ERS013251 |
| SN115 | 1995 | Vietnam | ERS013252 |
| SN116 | 2010 | Haiti | AELI00000000.1 |
| SN118 | 2004 | India | ERS013134 |
| SN119 | 1980 | India | ERS013145 |
| SN120 | 2007 | India | ERS013268 |
| SN121 | 1991 | Mozambique | ERS013181 |
| SN122 | 1994 | Bangladesh | ERS013278 |
| SN123 | 1992 | Colombia | ERS013175 |
| SN124 | 2007 | India | ERS013266 |
| SN125 | 1991 | India | ERS013132 |
| SN126 | 2001 | Bangladesh | ERS013238 |
| SN127 | 2007 | India | ERS013236 |
| SN128 (N16961) | 1975 | Bangladesh | AE003852/AE003853 |
| SN131 | 1979 | India | ERS013143 |
| SN132 | 2005 | India | ERS013239 |
| SN134 | 1979 | Bangladesh | ERS013250 |
| SN135 | 1985 | Kenya | ACHX00000000 |
| SN136 | 1992 | India | ERS013139 |
| SN138 | 2006 | Bangladesh | ERS013279 |
| SN140 | 2010 | Haiti | AELJ00000000.1 |
| SN141 | 2009 | Machakos | ERS013225 |
| SN144 | 1991 | Peru | ERS013275 |
| SN146 | 2007 | India | ERS013257 |
| SN147 | 2009 | India | ERS013147 |
| SN148 | 1957 | Indonesia | ERS013246 |
| SN149 | 1978 | Malaysia | ERS013141 |
| SN150 | 2009 | Machakos | ERS013221 |
| SN156 | 1999 | Bangladesh | ERS013260 |
| SN159 | 1991 | Peru | ERS013274 |
| SN161 | 1989 | India | ERS013178 |
| SN162 | 1980 | India | ERS013243 |
| SN163 | 2009 | Machakos | ERS013224 |
| SN164 | 2005 | Nairobi | ERS013212 |
| SN167 | 2001 | Bangladesh | ERS013259 |
| SN168 | 2007 | Kakuma | ERS013210 |
| SN170 | 2001 | Bangladesh | ERS013263 |
| SN171 | 2005 | Mozambique | ERS013241 |
| SN172 | 2010 | Haiti | CP003070.1/CP003069.1 |
| SN173 | 1989 | Vietnam | ERS013187 |

| | | | |
|---|---|---|---|
| SN175 | 2009 | Machakos | ERS013220 |
| SN176 | 2007 | India | ERS013240 |
| SN177 | 2004 | India | ERS013270 |
| SN178 | 1991 | Mozambique | ERS013180 |
| SN179 | 2009 | Tanzania | ERS013235 |
| SN181 | 2002 | Vietnam | ERS013253 |
| SN182 | 2006 | India | ERS013234 |
| SN183 | 2007 | Nairobi | ERS013217 |
| SN184 | 1991 | Mozambique | ERS013179 |
| SN185 | 1979 | Bangladesh | ERS013247 |
| SN186 | 2007 | Bangladesh | ERS013281 |
| SN187 | 2007 | India | ERS013267 |
| SN188 | 1978 | Bahrain | ERS013142 |
| SN189 | 2006 | India | ERS013255 |
| SN192 | 2006 | India | ERS013258 |
| SN194 | 1992 | Colombia | ERS013182 |
| SN197 | 2004 | Vietnam | ERS013271 |
| SN198 | 2007 | India | ERS013237 |
| SN200 | 2005 | Kakuma | ERS013211 |
| SN201 | 1991 | Peru | ERS013276 |
| SN202 | 1990 | India | ERS013131 |
| SN204 | 1973 | India | ERS013137 |
| SN205 | 1979 | India | ERS013146 |
| SN206 | 1994 | Bangladesh | ERS013126 |
| SN207 | 2000 | Bangladesh | ERS016137 |
| SN208 | 2001 | Bangladesh | ERS013261 |
| SN209 | 2004 | India | ERS013127 |
| SN210 | 2007 | Nairobi | ERS013218 |
| SN211 | 1989 | Angola | ERS013245 |
| SN212 | 2002 | Bangladesh | ACVW00000000 |
| SN213 | 2007 | India | ERS013269 |
| SN215 | 2010 | Pakistan | ERR051745 |
| SN216 | 2010 | Pakistan | ERR051746 |
| SN217 | 2010 | Pakistan | ERR051748 |
| SN218 | 2010 | Pakistan | ERR051749 |
| SN219 | 2010 | Pakistan | ERR051751 |
| SN220 | 2010 | Pakistan | ERR051752 |
| SN221 | 2010 | Pakistan | ERR051755 |
| SN222 | 2010 | Pakistan | ERR051756 |
| SN223 | 2010 | Pakistan | ERR051758 |
| SN224 | 2010 | Pakistan | ERR051759 |
| SN225 | 2010 | Pakistan | ERR051760 |
| SN226 | 2010 | Pakistan | ERR051761 |
| SN227 | 2010 | Pakistan | ERR051762 |
| SN228 | 2010 | Pakistan | ERR051763 |
| SN229 | 2010 | Pakistan | ERR051764 |
| SN230 | 2010 | Pakistan | ERR051765 |
| SN231 | 2010 | Pakistan | ERR051767 |

| | | | |
|---|---|---|---|
| SN232 | 2010 | Pakistan | ERR051768 |
| SN233 | 2010 | Pakistan | ERR051769 |
| SN234 | 2010 | Pakistan | ERR051770 |
| SN235 | 2010 | Pakistan | ERR051771 |
| SN236 | 2010 | Pakistan | ERR051772 |
| SN237 | 2010 | Pakistan | ERR051773 |
| SN238 | 2010 | Pakistan | ERR051775 |
| SN239 | 2010 | Pakistan | ERR051776 |
| SN240 | 2010 | Pakistan | ERR051777 |
| SN241 | 2010 | Pakistan | ERR051779 |
| SN242 | 2010 | Pakistan | ERR051780 |
| SN243 | 2010 | Pakistan | ERR051781 |
| SN244 | 2010 | Pakistan | ERR051782 |
| SN245 | 2010 | Pakistan | ERR051783 |
| SN246 | 2010 | Pakistan | ERR051784 |
| SN247 | 2010 | Pakistan | ERR051785 |
| SN248 | 2010 | Pakistan | ERR051786 |
| SN249 | 2010 | Pakistan | ERR051787 |
| SN250 | 2010 | Pakistan | ERR051788 |
| SN251 | 2010 | Pakistan | ERR051789 |
| SN252 | 2010 | Pakistan | ERR051790 |
| SN253 | 2010 | Nepal | SRX082797 |
| SN254 | 2010 | Nepal | SRX082833 |
| SN255 | 2010 | Nepal | SRX082835 |
| SN256 | 2010 | Nepal | SRX082836 |
| SN257 | 2010 | Nepal | SRX082837 |
| SN258 | 2010 | Nepal | SRX082838 |
| SN259 | 2010 | Nepal | SRX082839 |
| SN260 | 2010 | Nepal | SRX082840 |
| SN261 | 2010 | Nepal | SRX082841 |
| SN262 | 2010 | Nepal | SRX082842 |
| SN263 | 2010 | Nepal | SRX082843 |
| SN264 | 2010 | Nepal | SRX082938 |
| SN265 | 2010 | Nepal | SRX082939 |
| SN266 | 2010 | Nepal | SRX082940 |
| SN267 | 2010 | Nepal | SRX082941 |
| SN268 | 2010 | Nepal | SRX082942 |
| SN269 | 2010 | Nepal | SRX082943 |
| SN270 | 2010 | Nepal | SRX082944 |
| SN271 | 2010 | Nepal | SRX082945 |
| SN272 | 2010 | Nepal | SRX082946 |
| SN273 | 2010 | Nepal | SRX082947 |
| SN274 | 2010 | Nepal | SRX082948 |
| SN275 | 2010 | Nepal | SRX082949 |
| SN276 | 2010 | Nepal | SRX082834 |
| SN801 | 2011 | Togo | ERR572582 |
| SN802 | 2011 | Nigeria | ERR1878593 |
| SN803 | 2011 | Cameroon | ERR1878596 |

| | | | |
|---|---|---|---|
| SN804 | 2011 | Central African Republic | ERR1878603 |
| SN805 | 2011 | Cameroon | ERR1878597 |
| SN806 | 2011 | Democratic Republic of the Congo | ERR572781 |
| SN807 | 2011 | Ghana | ERR1024518 |
| SN808 | 2011 | Togo | ERR572589 |
| SN809 | 2011 | Togo | ERR572585 |
| SN810 | 2011 | Nigeria | ERR1878601 |
| SN811 | 2011 | Togo | ERR572840 |
| SN812 | 2011 | Nigeria | ERR1878594 |
| SN813 | 2011 | Central African Republic | ERR1878602 |
| SN814 | 2011 | Chad | ERR1878599 |
| SN815 | 2011 | Democratic Republic of the Congo | ERR572772 |
| SN816 | 2011 | Democratic Republic of the Congo | ERR572821 |
| SN817 | 2011 | Chad | ERR1878600 |
| SN818 | 2012 | Democratic Republic of the Congo | ERR386688 |
| SN819 | 2012 | Guinea | ERR386658 |
| SN820 | 2012 | Democratic Republic of the Congo | ERR386647 |
| SN821 | 2012 | Democratic Republic of the Congo | ERR386695 |
| SN822 | 2012 | Guinea | ERR386666 |
| SN823 | 2012 | Zambia | ERR386656 |
| SN824 | 2012 | Zambia | ERR386663 |
| SN825 | 2012 | Zambia | ERR386661 |
| SN826 | 2012 | Togo | ERR572842 |
| SN827 | 2012 | Democratic Republic of the Congo | ERR386711 |
| SN828 | 2012 | Democratic Republic of the Congo | ERR386712 |
| SN829 | 2012 | Democratic Republic of the Congo | ERR572548 |
| SN830 | 2012 | Democratic Republic of the Congo | ERR572807 |
| SN831 | 2012 | Togo | ERR572590 |
| SN832 | 2012 | Guinea Bissau | ERR1878605 |
| SN833 | 2012 | Guinea Bissau | ERR1878604 |
| SN834 | 2012 | Togo | ERR572592 |
| SN835 | 2012 | Guinea | ERR386704 |
| SN836 | 2013 | Democratic Republic of the Congo | ERR572559 |
| SN837 | 2013 | Democratic Republic of the Congo | ERR572836 |
| SN838 | 2013 | Democratic Republic of the Congo | ERR572563 |
| SN839 | 2013 | Democratic Republic of the Congo | ERR572837 |
| SN840 | 2014 | Ghana | ERR1024524 |
| SN841 | 2014 | Nigeria | ERR1878610 |
| SN842 | 2014 | Ghana | ERR1024521 |
| SN843 | 2014 | Democratic Republic of the Congo | ERR1878608 |
| SN844 | 2014 | Democratic Republic of the Congo | ERR1878607 |
| SN845 | 2014 | Nigeria | ERR1878609 |
| SN846 | 2014 | Ghana | ERR1024526 |
| SN847 | 2010 | India | ERR2269808 |
| SN848 | 2010 | India | ERR2269921 |

| | | | |
|---|---|---|---|
| SN849 | 2010 | India | ERR2269922 |
| SN850 | 2011 | India | ERR2269925 |
| SN851 | 2011 | Bangladesh | ERR2265590 |
| SN852 | 2011 | India | ERR2269923 |
| SN854 | 2011 | India | ERR2269924 |
| SN855 | 2012 | India | ERR2269928 |
| SN856 | 2012 | India | ERR2269927 |
| SN857 | 2012 | India | ERR2269929 |
| SN858 | 2012 | Iran | ERR2269835 |
| SN859 | 2013 | Iran | ERR2269837 |
| SN860 | 2013 | India | ERR2269945 |
| SN861 | 2013 | India | ERR2269930 |
| SN862 | 2013 | India | ERR2269944 |
| SN863 | 2013 | India | ERR2269946 |
| SN864 | 2013 | Iran | ERR2269836 |
| SN865 | 2014 | South Sudan | ERR2265646 |
| SN866 | 2014 | India | ERR2269949 |
| SN867 | 2014 | South Sudan | ERR2265645 |
| SN868 | 2014 | South Sudan | ERR2265591 |
| SN869 | 2014 | India | ERR2265649 |
| SN870 | 2014 | South Sudan | ERR2265648 |
| SN871 | 2014 | India | ERR2269948 |
| SN872 | 2014 | South Sudan | ERR2265647 |
| SN873 | 2014 | India | ERR2269947 |
| SN874 | 2015 | India | ERR2265652 |
| SN875 | 2015 | Iran | ERR2269838 |
| SN876 | 2015 | Iraq | ERR2265655 |
| SN877 | 2015 | Democratic Republic of the Congo | ERR2265650 |
| SN878 | 2015 | Iraq | ERR2265660 |
| SN879 | 2015 | Iraq | ERR2265658 |
| SN880 | 2015 | Democratic Republic of the Congo | ERR2265661 |
| SN881 | 2015 | Democratic Republic of the Congo | ERR2265651 |
| SN882 | 2015 | Iraq | ERR2265657 |
| SN883 | 2015 | India | ERR2269952 |
| SN884 | 2015 | India | ERR2269950 |
| SN885 | 2015 | Iraq | ERR2265659 |
| SN886 | 2015 | India | ERR2269953 |
| SN887 | 2015 | South Sudan | ERR2265654 |
| SN888 | 2015 | South Sudan | ERR2265653 |
| SN889 | 2015 | Iraq | ERR2265656 |
| SN890 | 2015 | India | ERR2269951 |
| SN891 | 2015 | Kenya | ERR2265589 |
| SN892 | 2016 | Yemen | ERR2265676 |
| SN893 | 2016 | India | ERR2270655 |
| SN894 | 2016 | South Sudan | ERR2265667 |
| SN895 | 2016 | Yemen | ERR2265675 |
| SN896 | 2016 | Yemen | ERR2269615 |

| | | | |
|---|---|---|---|
| SN897 | 2016 | South Sudan | ERR2265668 |
| SN898 | 2016 | Yemen | ERR2265674 |
| SN899 | 2016 | Yemen | ERR2265678 |
| SN900 | 2016 | Yemen | ERR2269613 |
| SN901 | 2016 | Yemen | ERR2269614 |
| SN902 | 2016 | Yemen | ERR2265677 |
| SN903 | 2016 | India | ERR2270656 |
| SN904 | 2016 | India | ERR2270657 |
| SN905 | 2016 | India | ERR2270658 |
| SN906 | 2017 | India | ERR2270661 |
| SN907 | 2017 | South Sudan | ERR2265671 |
| SN908 | 2017 | Yemen | ERR2269648 |
| SN909 | 2017 | Yemen | ERR2269715 |
| SN910 | 2017 | Yemen | ERR2269644 |
| SN911 | 2017 | Yemen | ERR2269809 |
| SN912 | 2017 | Yemen | ERR2269617 |
| SN913 | 2017 | South Sudan | ERR2265669 |
| SN914 | 2017 | Yemen | ERR2269714 |
| SN915 | 2017 | South Sudan | ERR2265672 |
| SN916 | 2017 | Yemen | ERR2269616 |
| SN917 | 2017 | Yemen | ERR2269716 |
| SN918 | 2017 | Yemen | ERR2269640 |
| SN919 | 2017 | South Sudan | ERR2265673 |
| SN920 | 2017 | Yemen | ERR2269643 |
| SN921 | 2017 | Yemen | ERR2269710 |
| SN922 | 2017 | Yemen | ERR2269650 |
| SN923 | 2017 | Yemen | ERR2269621 |
| SN924 | 2017 | Yemen | ERR2269709 |
| SN925 | 2017 | Yemen | ERR2269620 |
| SN926 | 2017 | Yemen | ERR2269834 |
| SN927 | 2017 | South Sudan | ERR2265670 |
| SN928 | 2017 | Yemen | ERR2269717 |
| SN929 | 2017 | Yemen | ERR2269811 |
| SN930 | 2017 | Yemen | ERR2269619 |
| SN931 | 2017 | Yemen | ERR2269647 |
| SN932 | 2017 | Yemen | ERR2269713 |
| SN933 | 2017 | Yemen | ERR2269622 |
| SN934 | 2017 | Yemen | ERR2269641 |
| SN935 | 2017 | Yemen | ERR2269711 |
| SN936 | 2017 | Yemen | ERR2269646 |
| SN937 | 2017 | India | ERR2270660 |
| SN938 | 2017 | Yemen | ERR2269833 |
| SN939 | 2017 | India | ERR2270662 |
| SN940 | 2017 | Yemen | ERR2269645 |
| SN941 | 2017 | Yemen | ERR2269718 |
| SN942 | 2017 | Yemen | ERR2269810 |
| SN943 | 2017 | Yemen | ERR2269642 |
| SN944 | 2017 | Yemen | ERR2269649 |

| | | | |
|---|---|---|---|
| SN945 | 2017 | Yemen | ERR2269712 |
| SN946 | 2017 | India | ERR2270659 |
| SN947 | 2017 | Yemen | ERR2269832 |
| SN948 | 2017 | Yemen | ERR2269618 |
| SN949 | 2018 | China | SAMN13544778 |
| SN950 | 2012 | Russia | SRX497591 |
| SN951 | 2010 | Russia | SRX498647 |
| SN953 | 2010 | India | ERS2159335 |
| SN954 | 2011 | China | SAMN36429565 |
| SN955 | 2011 | China | SAMN36429566 |
| SN956 | 2011 | China | SAMN36429567 |

**Table S3. Oligonucleotide primers used in this study.**

| Oligonucleotide designation | Sequence | Comment |
|---|---|---|
| ompW-T-F | CCGCTAAAAATGATGAAA | amplification of the *ompW* gene of *V. cholerae*, forward primer |
| ompW-T-R | TCCATCAAGTTTGTGTGA | amplification of the *ompW* gene of *V. cholerae*, reverse primer |
| pSRKGm-NdeI-ompW-F | TTTCACACAGGAAACAGCAATGAAACAAACCATTTGCGGCCT | construction of the target plasmid pSRKGm-ompW, forward primer |
| pSRKGm-KpnI-ompW-R | CACTATAGGGCGAATTGGGTACCTTAGAACTTATAACCACCCG | construction of the target plasmid pSRKGm-ompW, reverse primer |
| pSRKGm-ompW-2777F | GCGGCTATTTAACGACCCTGCC | amplification of the complementary plasmids with inserted *ompW* gene, forward primer |
| pSRKGm-ompW-3914R | GCCCAATACGCAAACCGCCTC | amplification of the complementary plasmids with inserted *ompW* gene, reverse primer |

**Table S4. Inferred migrations of PT6 strains between geographical locations in Sichuan Province.**

| Origins Destination | BT | EB | EMS | GL | HY | JKH | JY | LS | MG | MN | PZH | PG | WJ | XC | XD | YX | ZJ | Total |
|---|---|---|---|---|---|---|---|---|---|---|---|---|---|---|---|---|---|---|
| **BT** | 0 | 0 | 0 | 1 (2000) | 0 | 0 | 3 (1998) | 0 | 0 | 0 | 1 (1998) | 5 (1998) | 0 | 2 (1999) | 1 (1998) | 0 | 3(1998, 1999, 2000) | 16 |
| **EB** | 0 | 0 | 0 | 0 | 0 | 0 | 0 | 0 | 0 | 0 | 0 | 0 | 0 | 0 | 0 | 0 | 0 | 0 |
| **EMS** | 0 | 0 | 0 | 0 | 0 | 0 | 0 | 0 | 0 | 0 | 0 | 0 | 0 | 0 | 0 | 0 | 0 | 0 |
| **GL** | 0 | 1 (2000) | 0 | 0 | 2 (2000) | 2 (2000) | 2 (2001) | 1 (2000) | 2 (2000) | 0 | 0 | 0 | 1 (2000) | 1 (2000) | 0 | 0 | 0 | 12 |
| **HY** | 0 | 0 | 0 | 0 | 0 | 0 | 0 | 0 | 0 | 0 | 0 | 0 | 0 | 0 | 0 | 0 | 0 | 0 |
| **JKH** | 0 | 0 | 0 | 0 | 0 | 0 | 0 | 0 | 0 | 0 | 0 | 0 | 0 | 0 | 0 | 0 | 0 | 0 |
| **JY** | 1 (2001) | 0 | 0 | 0 | 0 | 0 | 0 | 0 | 0 | 0 | 0 | 0 | 0 | 0 | 0 | 0 | 0 | 1 |
| **LS** | 0 | 0 | 0 | 0 | 0 | 0 | 0 | 0 | 0 | 0 | 0 | 0 | 0 | 0 | 0 | 0 | 0 | 0 |
| **MG** | 0 | 0 | 0 | 0 | 0 | 0 | 0 | 0 | 0 | 0 | 0 | 0 | 0 | 0 | 0 | 0 | 0 | 0 |
| **MN** | 0 | 0 | 0 | 0 | 0 | 0 | 0 | 0 | 0 | 0 | 0 | 0 | 0 | 0 | 0 | 0 | 0 | 0 |
| **PZH** | 0 | 0 | 0 | 0 | 0 | 0 | 0 | 0 | 0 | 0 | 0 | 0 | 0 | 0 | 0 | 0 | 0 | 0 |
| **PG** | 0 | 0 | 0 | 0 | 0 | 0 | 0 | 0 | 0 | 0 | 0 | 0 | 0 | 1 (1999) | 0 | 0 | 0 | 1 |
| **WJ** | 0 | 0 | 3 (2000) | 1 (2000) | 0 | 1 (2000) | 0 | 0 | 0 | 0 | 0 | 0 | 0 | 0 | 0 | 0 | 0 | 5 |
| **XC** | 0 | 0 | 0 | 1 (2000) | 0 | 0 | 0 | 0 | 0 | 1 (1999) | 0 | 1 (2000) | 0 | 0 | 0 | 1 (1999) | 0 | 4 |

| | | | | | | | | | | | | | | | | | | |
|---|---|---|---|---|---|---|---|---|---|---|---|---|---|---|---|---|---|---|
| **XD** | 0 | 0 | 0 | 0 | 0 | 0 | 0 | 0 | 0 | 0 | 0 | 0 | 0 | 0 | 0 | 0 | 0 | 0 |
| **YX** | 0 | 0 | 0 | 0 | 0 | 0 | 0 | 0 | 0 | 0 | 0 | 0 | 0 | 0 | 0 | 0 | 0 | 0 |
| **ZJ** | 0 | 0 | 0 | 0 | 0 | 0 | 0 | 0 | 0 | 0 | 0 | 1 (2000) | 0 | 0 | 0 | 0 | 0 | 1 |
| **Total** | 1 | 1 | 3 | 3 | 2 | 3 | 5 | 1 | 2 | 1 | 1 | 7 | 1 | 4 | 1 | 1 | 3 | 40 |
